# Rapid development of myeloproliferative neoplasm in mice with *Ptpn11*^*D61Y*^ mutation and haploinsufficient for *Dnmt3a*

**DOI:** 10.18632/oncotarget.23680

**Published:** 2017-12-26

**Authors:** Lisa Deng, Briana M. Richine, Elizabeth L. Virts, Victoria N. Jideonwo-Auman, Rebecca J. Chan, Reuben Kapur

**Affiliations:** ^1^Herman B Wells Center for Pediatric Research, Indiana University School of Medicine, Indianapolis, IN, USA; ^2^Department of Medical and Molecular Genetics, Indiana University School of Medicine, Indianapolis, IN, USA; ^3^Department of Microbiology and Immunology, Indiana University School of Medicine, Indianapolis, IN, USA; ^4^Department of Pediatrics, Indiana University School of Medicine, Indianapolis, IN, USA; ^5^Gilead Sciences, Inc., Foster City, CA, USA

**Keywords:** AML, JMML, DNMT3A, SHP2, myeloproliferation

## Abstract

*PTPN11* gain-of-function mutation is the most common mutation found in patients with juvenile myelomonocytic leukemia and DNMT3A loss occurs in over 20% of acute myeloid leukemia patients. We studied the combined effect of both *Ptpn11* gain-of-function mutation (D61Y) and *Dnmt3a* haploinsufficiency on mouse hematopoiesis, the presence of which has been described in both juvenile myelomonocytic leukemia and acute myeloid leukemia patients. Double mutant mice rapidly become moribund relative to any of the other genotypes, which is associated with enlargement of the spleen and an increase in white blood cell counts. An increase in the mature myeloid cell compartment as reflected by the presence of Gr1^+^Mac1^+^ cells was also observed in double mutant mice relative to any other group. Consistent with these observations, a significant increase in the absolute number of granulocyte macrophage progenitors (GMPs) was seen in double mutant mice. A decrease in the lymphoid compartment including both T and B cells was noted in the double mutant mice. Another significant difference was the presence of extramedullary erythropoiesis with increased erythroid progenitors in the spleens of *Dnmt3a*^*+/*−^*;D61Y* mice relative to other groups. Taken together, our results suggest that the combined haploinsufficiency of *Dnmt3a* and presence of an activated *Shp2* changes the composition of multiple hematopoietic lineages in mice relative to the individual heterozygosity of these genes.

## INTRODUCTION

Loss of DNA methyltransferase 3A (DNMT3A) activity has been shown to lead to hematopoietic stem cell differentiation defects and the development of myeloid malignancies. *DNMT3A* is commonly mutated in myeloid diseases, with mutations found in over 20% of all acute myeloid leukemia (AML) patients [1], in 8% of myelodysplastic syndrome (MDS) patients [2], and in smaller frequencies in other leukemias. For AML patients, 60% of those with a *DNMT3A* mutation are heterozygous at Arginine 882 (R882), a dominant negative mutation that results in less than 80% protein activity [3]. The remaining patients usually demonstrate a compound heterozygous or homozygous mutation.

In contrast to AML, individuals with clonal hematopoiesis commonly bear a loss-of-function mutation in only one copy of *DNMT3A* [4]. In this pre-leukemic state, almost all *DNMT3A* mutations are nonsynonymous, truncating, or splicing, and mutations in R882 are rare [5]. These otherwise healthy individuals are at an increased risk of developing MDS/AML due to acquisition of a second driver mutation [6] and tend to have a poor overall prognosis [1]. Thus, although DNMT3A protein function must be almost completely lost in order to cause malignancy [7], heterozygous loss-of-function mutations are often found combined with a driver mutation in the development of frank leukemia. Furthermore, *Dnmt3a* haploinsufficiency alone with no other lesions is sufficient for mice to develop myeloid malignancies when aged to 18–24 months [8].

Here, we studied the effects of *Dnmt3a* haploinsufficiency combined with *Ptpn11*^D61Y^, a gain-of-function mutation in the protein tyrosine phosphatase SHP2, which is the most commonly mutated gene in juvenile myelomonocytic leukemia (JMML) and is used as a mouse model of myeloproliferative disease [9]. Concurrent mutations in both *DNMT3A* and *PTPN11*, albeit rare, have been reported in AML [10, 11] and in JMML patients [12]. JMML is an aggressive myeloproliferative neoplasm (MPN) of early childhood with no effective chemotherapeutic treatment and a poor prognosis.

## RESULTS

**Figure 1:**
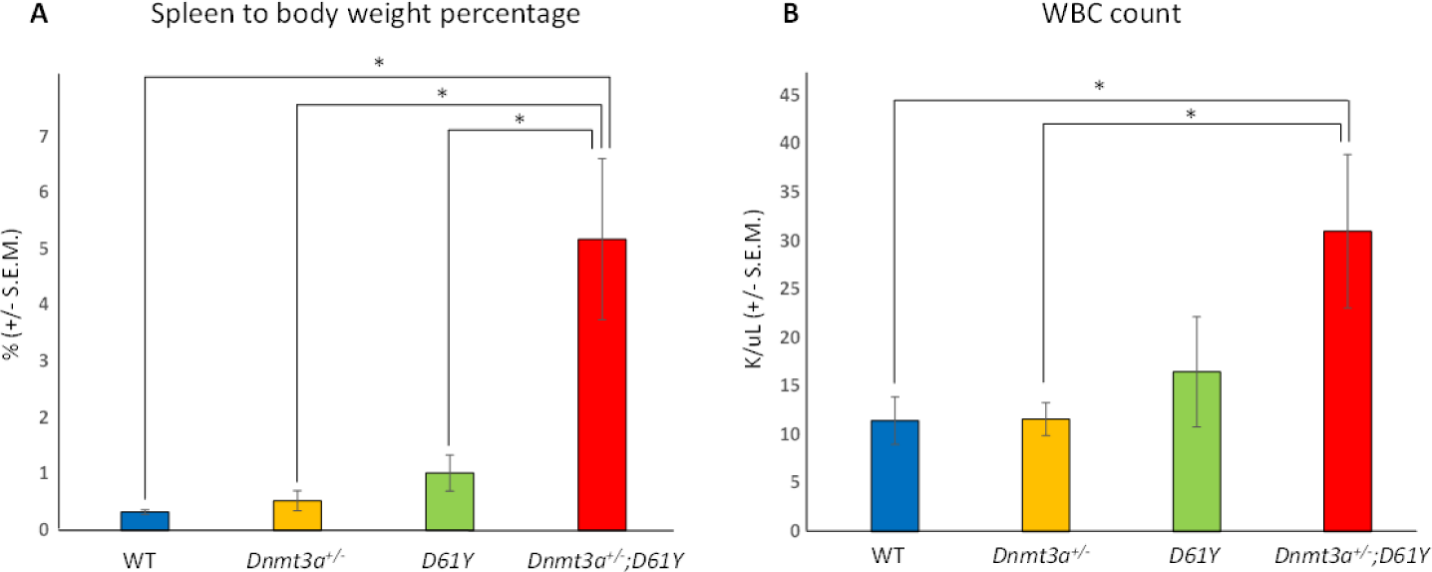
*Dnmt3a*^*+/*−^*;D61Y* mice show splenomegaly and leukocytosis at the time of death (**A**) Average spleen to body weight ratio of mice at the time of euthanasia; *n* = 13 for WT, *n* = 9 for *Dnmt3a*^*+/−*^*, n* = 10 for *D61Y*, *n* = 6 for *Dnmt3a*^*+/−*^*;D61Y*, ^*^p < 0.0001 comparing *Dnmt3a*^*+/−*^*;D61Y* to WT, ^*^p = 0.003 comparing *Dnmt3a*^*+/−*^*;D61Y* to *Dnmt3a*^*+/−*^, ^*^p = 0.003 comparing *Dnmt3a*^*+/−*^*;D61Y* to *D61Y*; statistical analyses performed by unpaired, two-tailed, Student’s *t*-test. (**B**) Average WBC count in peripheral blood of mice immediately prior to euthanasia; *n* = 8 for WT, *n* = 7 for *Dnmt3a*^*+/−*^, *n* = 5 for *D61Y*, *n* = 5 for *Dnmt3a*^*+/−*^*;D61Y*; ^*^p = 0.0157 comparing *Dnmt3a*^*+/−*^*;D61Y* to WT, ^*^p = 0.018 comparing *Dnmt3a*^*+/−*^*;D61Y* to *Dnmt3a*^*+/−*^; statistical analyses performed by unpaired, two-tailed, Student’s *t*-test.

We crossed *Ptpn11*^*D61Y/+*^;Mx1-*Cre*^*+*^ mice [9] with *Dnmt3a*^*+/−*^ mice [13] to produce Mx1-*Cre*^*-*^ (WT), *Dnmt3a*^*+/−*^*;Ptpn11*^*+/+*^*;*Mx1-*Cre*^*+*^ (*Dnmt3a*^*+/−*^)*, Dnmt3a*^*+/+*^*;Ptpn11*^*D61Y/+*^*;*Mx1-*Cre*^*+*^ (*D61Y*), and *Dnmt3a*^*+/−*^*;Ptpn11*^*D61Y/+*^*;*Mx1-*Cre*^*+*^ (*Dnmt3a*^*+/−*^*;D61Y*) mice. Six cohorts of mice, with 1 to 4 mice per genotype, were treated with polyI:polyC to knockout one copy of *Dnmt3a* and to knock-in the mutant *Ptpn11*^*D61Y*^ allele. All mice were followed until the *Dnmt3a*^*+/−*^*;D61Y* mice became moribund and the entire cohort was euthanized for analysis, which occurred at an average of 24 weeks after polyI:polyC treatment. We found that at the time when the *Dnmt3a*^*+/−*^*;D61Y* mice appeared moribund (thin, hunched, increased respiratory rate and effort, abdominal distension, ruffled fur, pale extremities), the Mx1-*Cre*^−^ or single mutant mice of the same cohort remained healthy. Upon euthanasia, the *Dnmt3a*^*+/−*^*;D61Y* mice showed obvious splenomegaly and the spleen to body weight percentage was significantly increased compared to the other three genotypes (Figure 1A). In addition to splenomegaly, the double mutant mice also showed significantly higher peripheral blood WBC counts compared to WT or *Dnmt3a*^*+/−*^ mice (Figure 1B).

**Figure 2:**
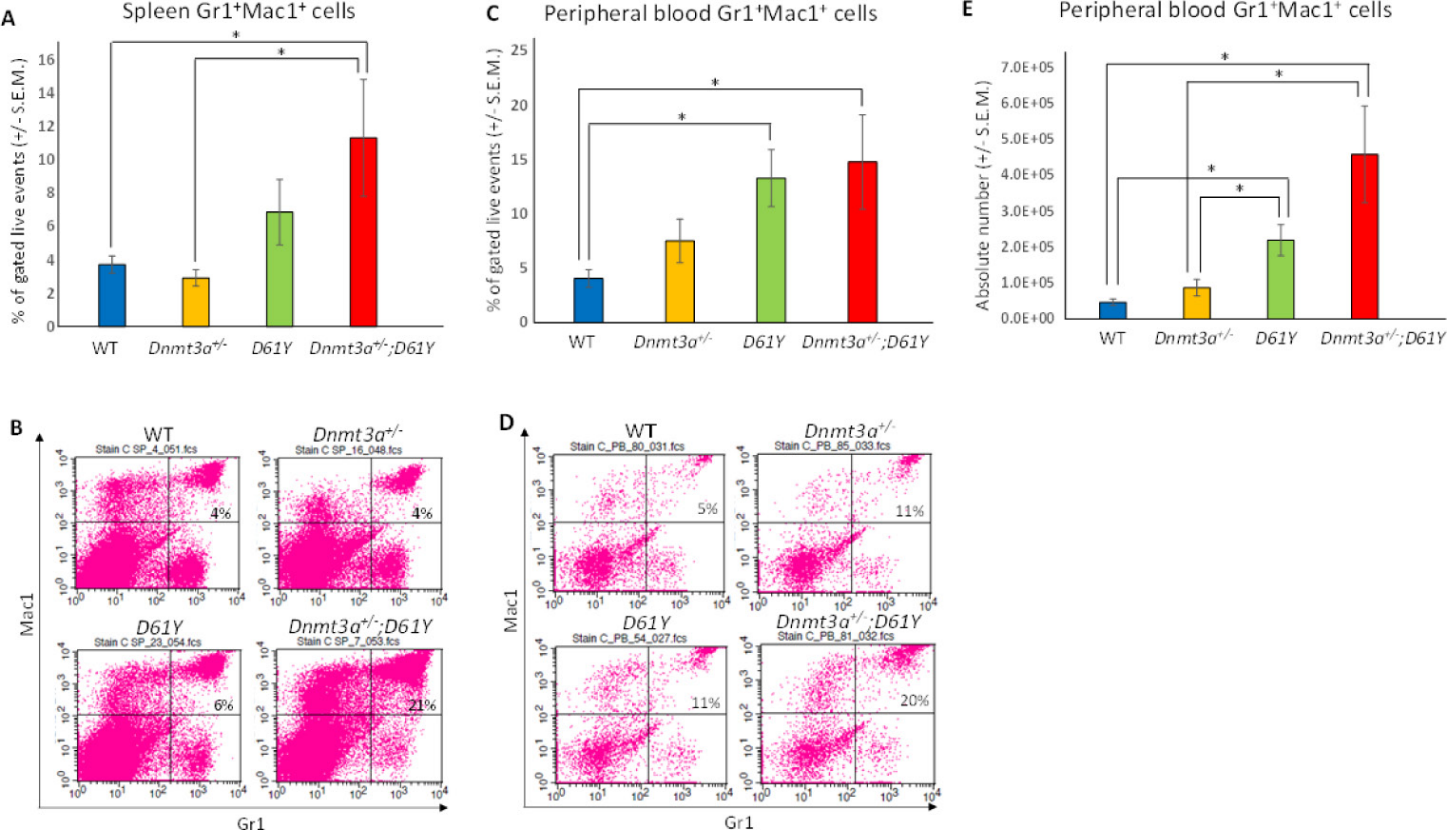
*Dnmt3a*^*+/*−^*;D61Y* mice have increased myeloid cells in the periphery at the time of death (**A**) Spleen average percentage of myeloid cells (Gr1^+^Mac1^+^) gated on live events and (**B**) representative flow diagrams; *n* = 10 for WT, *n* = 7 for *Dnmt3a*^*+/−*^, *n* = 9 for *D61Y*, *n* = 5 for *Dnmt3a*^*+/−*^*;D61Y*, ^*^p = 0.0095 comparing *Dnmt3a*^*+/−*^*;D61Y* to WT, ^*^p = 0.0177 comparing *Dnmt3a*^*+/−*^*;D61Y* to *Dnmt3a*^*+/−*^; statistical analyses performed by unpaired, two-tailed, Student’s *t*-test. (**C**) Peripheral blood average percentage of myeloid cells (Gr1^+^Mac1^+^) gated on live events and (**D**) representative flow diagrams; *n* = 12 for WT, *n* = 9 for *Dnmt3a*^*+/−*^, *n* = 9 for *D61Y*, *n* = 5 for *Dnmt3a*^*+/−*^*;D61Y*, ^*^p = 0.0025 comparing *Dnmt3a*^*+/−*^*;D61Y* to WT, ^*^p = 0.0009 comparing *D61Y* to WT; statistical analyses performed by unpaired, two-tailed, Student’s *t*-test. (**E**) Peripheral blood average absolute number of myeloid cells (Gr1^+^Mac1^+^) calculated by multiplying percentage and WBC count; *n* = 12 for WT, *n* = 9 for *Dnmt3a*^*+/−*^, *n* = 9 for *D61Y*, *n* = 5 for *Dnmt3a*^*+/−*^*;D61Y*, ^*^p = 0.0002 comparing *Dnmt3a*^*+/−*^*;D61Y* to WT, ^*^p = 0.0034 comparing *Dnmt3a*^*+/−*^*;D61Y* to *Dnmt3a*^*+/−*^*,* ^*^p = 0.0003 comparing *D61Y* to WT, ^*^p = 0.0159 comparing *D61Y* to *Dnmt3a*^*+/−*^*;* statistical analyses performed by unpaired, two-tailed, Student’s *t*-test.

**Figure 3:**
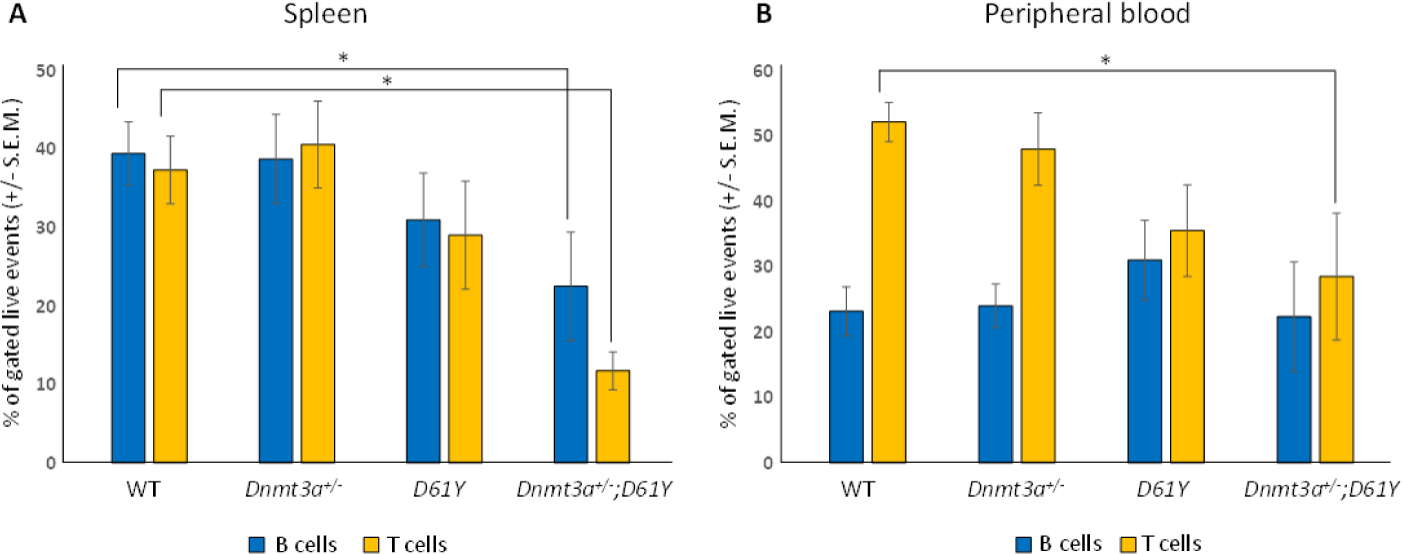
*Dnmt3a*^*+/*−^*;D61Y* mice have decreased B and T cells in the periphery at the time of death (**A**) Spleen average percentage of B cells (CD19^+^ or B220^+^) and T cells (CD4^+^ or CD8^+^) gated on live events; *n* = 13 for WT, *n* = 9 for *Dnmt3a*^*+/−*^, *n* = 9 for *D61Y*, *n* = 6 for *Dnmt3a*^*+/−*^*;D61Y*, ^*^p = 0.0386 comparing *Dnmt3a*^*+/−*^*;D61Y* to WT B cells, ^*^p = 0.0013 comparing *Dnmt3a*^*+/−*^*;D61Y* to WT T cells; statistical analyses performed by unpaired, two-tailed, Student’s *t*-test. (**B**) Peripheral blood average percentage of B cells (CD19^+^ or B220^+^) and T cells (CD4^+^ or CD8^+^) gated on live events; *n* = 12 for WT, *n* = 9 for *Dnmt3a*^*+/−*^, *n* = 9 for *D61Y*, *n* = 5 for *Dnmt3a*^*+/−*^*;D61Y*, ^*^p = 0.0071 comparing *Dnmt3a*^*+/−*^*;D61Y* to WT T cells; statistical analyses performed by unpaired, two-tailed, Student’s *t*-test.

Flow cytometric analysis of the spleen and peripheral blood to assess the frequency of Gr1^+^Mac1^+^ myeloid cells revealed a significantly higher percentage of these mature myeloid cells in the *Dnmt3a*^*+/−*^*;D61Y* mice compared to WT and *Dnmt3a*^*+/−*^ mice in the spleen, and compared to WT mice in the peripheral blood (Figure 2A–2D). In contrast, the myeloid cell frequencies in the single mutant mice were not statistically different from WT mice except for *D61Y* mice in the peripheral blood (Figure 2C and 2D). The absolute number of Gr1^+^Mac1^+^ cells were also significantly greater in double mutant mice relative to the WT and *Dnmt3a*^*+/−*^ mice (Figure 2E). In contrast to the increase in myeloid cells, the T and B cells were decreased in the spleen and peripheral blood of *Dnmt3a*^*+/−*^*;D61Y* mice relative to other groups (Figure 3A and 3B).

**Figure 4:**
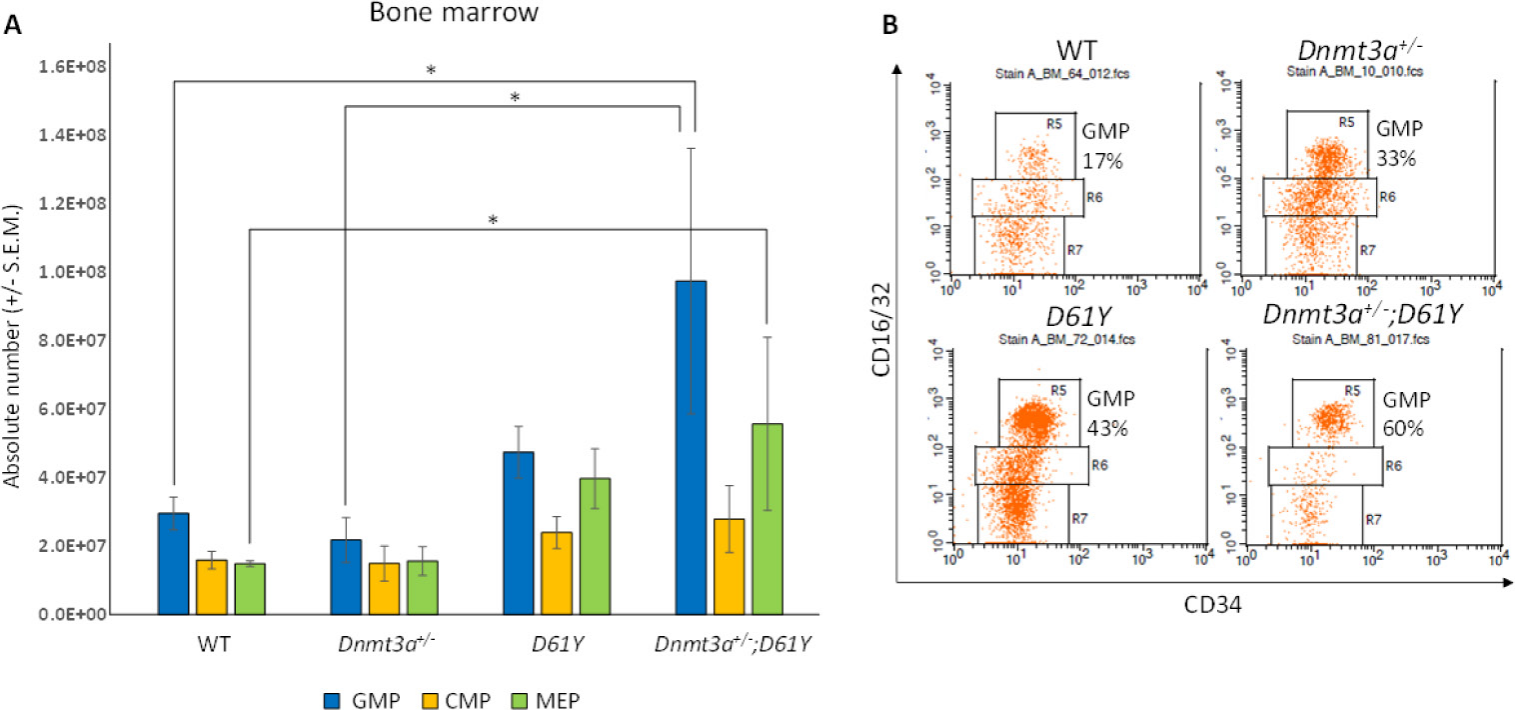
*Dnmt3a*^*+/*−^*;D61Y* mice have increased GMPs and MEPs in the bone marrow at the time of death (**A**) Absolute numbers of granulocyte monocyte progenitors (GMPs), common myeloid progenitors (CMPs), and megakaryocyte erythrocyte progenitors (MEPs) in bone marrow, gated on lineage^−^cKit^+^Sca1^−^ events and (**B**) representative flow diagrams; *n* = 10 for WT, *n* = 6 for *Dnmt3a*^*+/−*^, *n* = 6 for *D61Y*, *n* = 4 for *Dnmt3a*^*+/−*^*;D61Y*, ^*^p = 0.0159 comparing *Dnmt3a*^*+/−*^*;D61Y* GMPs to WT GMPs, ^*^p = 0.0445 comparing *Dnmt3a*^*+/−*^*;D61Y* GMPs to *Dnmt3a*^*+/−*^ GMPs, ^*^p = 0.0186 comparing *Dnmt3a*^*+/−*^*;D61Y* MEPs to WT MEPs; statistical analyses performed by unpaired, two-tailed, Student’s *t*-test.

**Figure 5:**
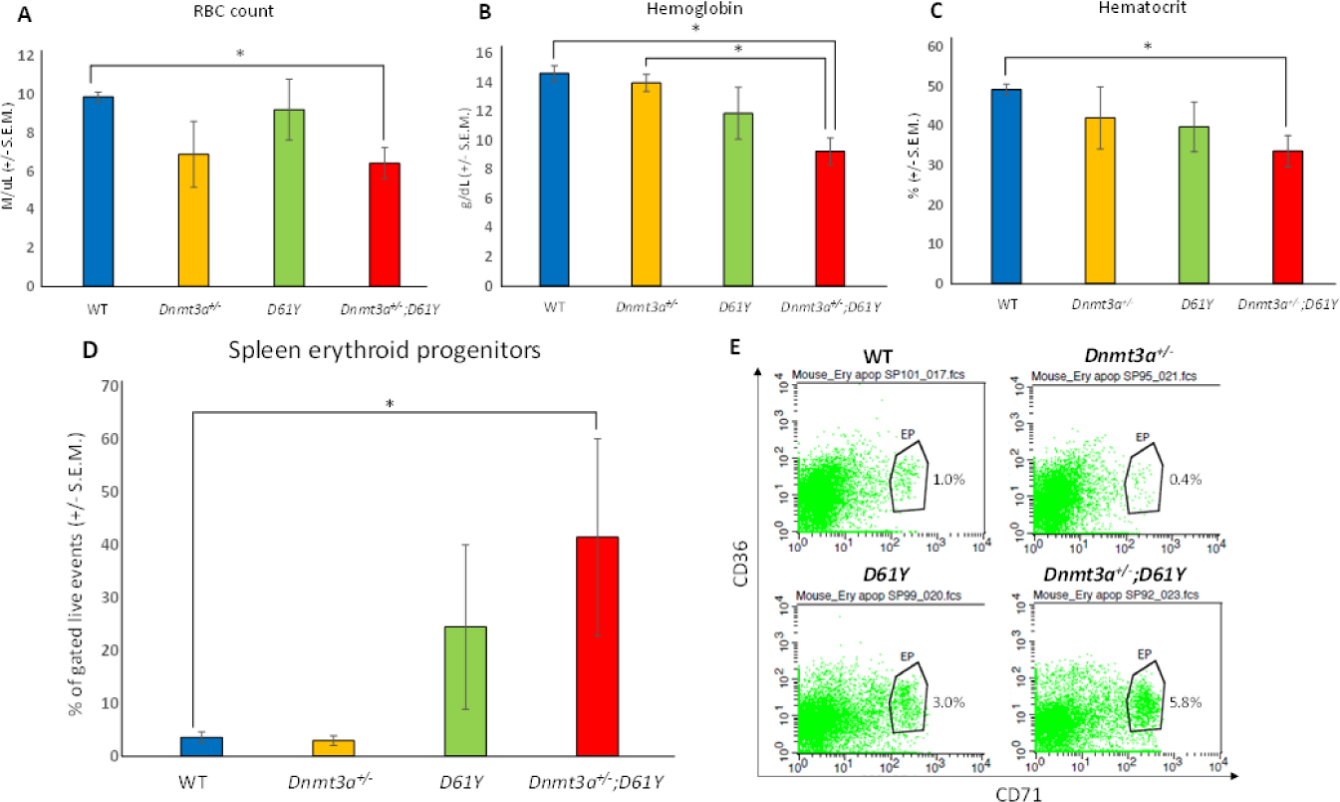
*Dnmt3a*^*+/*−^*;D61Y* mice have anemia with compensatory erythropoiesis in spleen (**A**) Average peripheral blood red blood cell (RBC) count in mice immediately prior to euthanasia; *n* = 8 for WT, *n* = 7 for *Dnmt3a*^*+/−*^, *n* = 5 for *D61Y*, *n* = 5 for *Dnmt3a*^*+/−*^*;D61Y*, ^*^p = 0.0005 comparing *Dnmt3a*^*+/−*^*;D61Y* to WT. (**B**) Average peripheral blood hemoglobin values in mice immediately prior to euthanasia; *n* = 8 for WT, *n* = 6 for *Dnmt3a*^*+/−*^, *n* = 5 for *D61Y*, *n* = 5 for *Dnmt3a*^*+/−*^*;D61Y*, ^*^p = 0.0002 comparing *Dnmt3a*^*+/−*^*;D61Y* to WT, ^*^p = 0.0016 comparing *Dnmt3a*^*+/−*^*;D61Y* to *Dnmt3a*^*+/−*^. (**C**) Average peripheral blood hematocrit values in mice immediately prior to euthanasia; *n* = 8 for WT, *n* = 7 for *Dnmt3a*^*+/−*^, *n* = 5 for *D61Y*, *n* = 5 for *Dnmt3a*^*+/−*^*;D61Y*, ^*^p = 0.001 comparing *Dnmt3a*^*+/−*^*;D61Y* to WT, statistical analyses performed by unpaired, two-tailed, Student’s *t*-test. (**D**) Average percentage of erythroid progenitors (CD36^+^CD71^+^) in spleen gated on live events, with (**E**) representative flow diagrams; *n* = 6 for WT, *n* = 4 for *Dnmt3a*^*+/*−^, *n* = 3 for *D61Y*, *n* = 3 for *Dnmt3a*^*+/−*^*;D61Y*; ^*^p = 0.0177 comparing *Dnmt3a*^*+/−*^*;D61Y* to WT, statistical analyses performed by unpaired, two-tailed, Student’s *t*-test.

In an effort to explain the increase in mature myeloid cells in the double mutant mice, we performed flow cytometric analysis on bone marrow cells from all four genotypes to assess the number of granulocyte macrophage progenitors (GMPs). As seen in Figure 4, *Dnmt3a*^*+/−*^*;D61Y* mice showed a significant increase in the absolute number of GMPs compared to WT and *Dnmt3a*^*+/−*^ mice. The double mutant mice also had significantly more absolute numbers of megakaryocyte erythrocyte progenitors (MEPs) compared to WT mice, but no significant differences in common myeloid progenitors (CMPs) were observed among any of the four groups (Figure 4). The double mutant mice also showed signs of anemia, with significant decreases in peripheral red blood cell counts, hemoglobin levels, as well as hematocrits relative to WT mice (Figure 5A–5C). Consistent with these observations, within the erythroid lineage, the CD36^+^CD71^+^ erythroid progenitors (EPs) were significantly increased in the spleens of the *Dnmt3a*^*+/−*^*;D61Y* mice, suggesting compensatory erythropoiesis (Figure 5D and 5E). Although EP frequency was not increased in the *Dnmt3a*^*+/−*^*;D61Y* bone marrow compartment (data not shown), the increased splenic erythropoiesis may explain the increased bone marrow MEP numbers.

## DISCUSSION

AML patients often have complete loss of *DNMT3A* enzyme activity, but its role when only partially inhibited and in combination with a second driver mutation of leukemia is not known. Here we show that mice with heterozygous loss of *Dnmt3a* combined with a gain-of-function Shp2 mutation, *D61Y*, develop more rapid disease progression and earlier mortality. Mice expressing Shp2D61Y do not usually succumb to leukemia until 45 weeks after induction of expression [9] and mice with *Dnmt3a* haploinsufficiency do not develop disease until approximately 80 weeks [8]. We found that mice with the two mutations together become moribund much earlier at 24 weeks, indicating that they cooperate to promote myeloid leukemia progression and to shorten survival.

The most marked phenotypic changes we observed in the *Dnmt3a*^*+/−*^*;D61Y* mice were splenomegaly, mature myeloid cell expansion in the periphery as well as increased GMPs in the bone marrow. These mice also exhibited signs of anemia, perhaps due to defects in erythrocyte cell maturation in the bone marrow. In support of this notion, MEPs in the bone marrow of compound mutant mice were increased and there were more erythroid progenitors present in the spleen, which most likely contributed to the splenomegaly in these mice.

The disease course of JMML also exhibits pronounced splenomegaly, sometimes even in the absence of highly elevated WBC count. It has been previously reported that mice with *Dnmt3a*^−*/*−^ combined with *Kras*^*G12D/+*^ mutation, another common mutation found in JMML patients, developed stress erythropoiesis in the spleen [14]. Perhaps one reason JMML patients develop extreme splenomegaly is compensatory splenic erythropoiesis, as observed in the *Dnmt3a*^*+/−*^*;D61Y* mice in this study.

Because *DNMT3A* and *PTPN11* mutations are found in combination in AML and JMML patients, our double mutant mice provide a novel clinically relevant model for developing and evaluating therapies for myeloid leukemia. In AML, the presence of a *DNMT3A* mutation led to significantly shortened overall survival [1]. For JMML, a disease with poor prognosis, there is currently no established decision-making process for determining which patients would be candidates for experimental therapies. However, it is known that the number of mutations present at diagnosis is strongly correlated with survival when comparing patients who have 0–1 mutations to those with 2 or more mutations [12]. Further work must be done to find the importance of having specifically the *DNMT3A* and *PTPN11* genes mutated together, but these are two commonly mutated genes in myeloid leukemia that we have now shown leads to accelerated disease.

The mechanism of how *Dnmt3a* loss cooperates with driver mutations to accelerate disease progression is still unknown, but our *Dnmt3a*^*+/−*^*;D61Y* mouse model will be useful to explore this question. We speculate that the changes in methylation caused by reduced DNMT3A activity may lead to epigenetic changes that alter the normal transcription of tumor suppressor genes needed to dampen Shp2 signaling. Future work doing a genome-wide transcriptional analysis would help to elucidate the mechanism of enhanced myeloid leukemia observed in the double mutant mice.

## METHODS

### Animal husbandry

Mice with a conditional mutant *Shp2* allele, *LSL-Shp2*^*D61Y/+*^, have been previously described [9]. Mice with a conditional knockout *Dnmt3a* allele have been previously described [13]. Expression of the *D61Y* mutation, the *Dnmt3a* mutation, and Mx1-*cre* were confirmed by genotyping. Three intraperitoneal injections of 300ug polyI:polyC were administered concurrently to mice of each cohort. Mice were housed and bred in accordance with the Institutional Animal Care and Use Committee of the Indiana University School of Medicine.

### Flow cytometry

Cell suspensions were incubated for 5 minutes with 10% rat serum (MP Biomedicals) and 0.2% BSA (Roche) in PBS, then stained for 30 minutes at 4°C with biotinylated lineage markers (Mac1, Gr1, CD4, CD8, B220, Ter119, IL7Rα, CD19, and CD3 with streptavidin PerCPcy5.5 as secondary), anti-Sca1-PE, anti-cKit-APC, anti-CD34-FITC, anti-CD16/32-PEcy7, anti-Gr1-FITC, anti-Mac1-APC, biotinylated anti-CD4 and anti-CD8 (with streptavidin APC as secondary), anti-B220-PE, anti-CD71-PE, and/or anti-CD36-APC (eBioscience and BD Biosciences). The analyzer used for flow cytometry was the BD LSR II and data was analyzed using CellQuest.

### Complete blood counts

Peripheral blood was collected from the saphenous vein of mice and complete blood counts were measured using a Hemavet 950 (Drew Scientific Group).

### Statistical calculations

GraphPad was used to perform the unpaired, two-tailed, Student’s *t*-tests.

## Abbreviations

*DNMT3A*: DNA methyltransferase 3A
AML: acute myeloid leukemia
MDS: myelodysplastic syndrome
JMML: juvenile myelomonocytic leukemia
MPN: myeloproliferative neoplasm
GMP: granulocyte monocyte progenitor
CMP: common myeloid progenitor
MEP: megakaryocyte erythrocyte progenitor
EP: erythroid progenitor
